# Pyrogen reaction and conversion of sustained ventricular tachycardia to sinus rhythm: two case reports

**DOI:** 10.1186/1757-1626-2-6637

**Published:** 2009-04-03

**Authors:** Quan-Zhou Feng, Xiao-Jun Ma

**Affiliations:** 1 Institute of Geriatric Cardiology, Chinese PLA General Hospital, Fuxing Road 28, Beijing, 100853, China; 2 Department of Internal Medicine, People's Hospital of Wuzhi County, Xinghua Road 120, Wuzhi County, 454950, Henan Province, China

## Abstract

**Introduction:**

Pyrogen reaction is a side effect of intravenous infusion of solution on body; sustained ventricular tachycardia is a serious arrhythmia, no relationship between them has been reported before.

**Case presentation:**

Two patients with sustained ventricular tachycardia refractory to lidocaine happened to have pyrogen reaction. The sustained ventricular tachycardia was converted to sinus rhythm after the pyrogen reaction.

**Conclusion:**

The conversion of sustained ventricular tachycardia might be related to pyrogen reaction. The effects of pyrogen reaction on sustained ventricular tachycardia need further research.

## Introduction

Pyrogen reaction is a febrile phenomenon caused by infusion of solution contaminated, and commonly manifested by cold, chill and fever [1]. With improved sterilization and generalized application of infusion set (single-use), the prevalence of pyrogen reaction has been controlled, but still exists in clinical practice. Sustained ventricular tachycardia (three or more consecutive ventricular complexes that last more than 30 seconds) is a serious arrhythmia, should be converted with medical therapy or defibrillator [2,3]. But when sustained ventricular tachycardia was refractory to medical therapy and happened to be followed by pyrogen reaction, the arrhythmia was surprisingly converted to sinus rhythm after the reaction, which is really rare.

We describe two patients with sustained ventricular tachycardia refractory to lidocaine, an only available drug, who were converted to sinus rhythm after pyrogen reaction.

## Case presentation

### Case report 1

**Figure 1:**
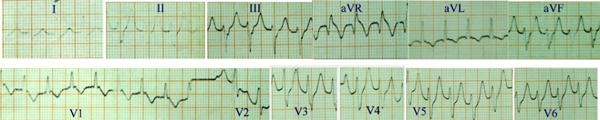
**Electrocardiogram of case1: Ventricular tachycardia, ventricular rate 180 beats per minute, was refractory to lidocaine**.

A 70-year-old Chinese man was admitted to hospital on July 13, 1985, because of palpitations for 8 hours. He had a four-time history of hospitalization because of sustained ventricular tachycardia, which was converted to sinus rhythm with lidocaine. Physical examination was normal apart from heart rate of 180 beats per minute. Serum electrolytes were normal. An electrocardiogram showed ventricular tachycardia. He was intravenously administered with 100 mg of lidocaine in 40 ml of 25% glucose for three times at interval of 10 min and converted to sinus rhythm. Three days later, his ventricular tachycardia recurred and heart rate was 180 beats per min (Figure 1). After administration of 100 mg of lidocaine in 40 ml of 25% glucose intravenously for four times at an interval of 10 minutes, the patient did not recover from ventricular tachycardia. As no other antiarrhythmic agents and defibrillator were available in the hospital, intravenous infusion of 5% glucose solution was slowly administrated. Thirty minutes later, pyrogen reaction happened, and he then began to feel cold and chill. The infusion was immediately stopped and 25 mg promethazine was intramuscularly injected at once. 20 minutes later, the symptoms stopped, his temperature increased to 39.5°C. His cardiac rhythm reverted to sinus rhythm, and his heart rate reduced to 92 beats per minute.

### Case report 2

**Figure 2:**
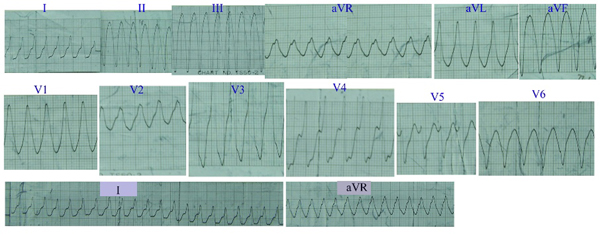
**Electrocardiogram of case 2: Ventricular tachycardia, ventricular rate 200 beats per minute, was refractory to lidocaine**.

A 62-year-old Chinese man was admitted to hospital on July 25, 1985, because of recurrent palpitations and shortness of breath. On examination, there was an increased respiratory rate. A raised jugular venous pressure was noted, together with hepatomegaly and marked peripheral edema. His pulse was 94 beats per minute and regular. Blood pressure was 90/10 mmHg. The apex beat was displaced laterally. Auscultation revealed a grade IV/IV systolic murmur, a harsh low-pitched diastolic murmur at the apex and fine rales at the lung bases. An electrocardiogram showed sinus rhythm. A chest radiograph revealed cardiomegaly and pulmonary venous congestion. Serum potassium, sodium, calcium and magnesium were normal. The patient was diagnosed as congestive heart failure, rheumatic heart disease: mitral stenosis and insufficiency, and was treated with inhalation of oxygen, vasodilator, diuretics and antibiotics. Two days later, his condition improved. On the third hospital day, the patient's palpitation recurred. Heart rate was 180 beats per min. The electrocardiogram revealed ventricular tachycardia. After administration of 100 mg of lidocaine in 40 ml of 25% glucose intravenously, the ventricular tachycardia was reverted to sinus rhythm. Two hours later, the ventricular tachycardia recurred (Figure 2). Heart rate increased up to 200 beats per min. Even though, 100 mg of lidocaine in 40 ml of 25% glucose being intravenously administered for four times at interval of 10 minutes, it failed to convert ventricular tachycardia to sinus rhythm. Blood pressure dropped to zero. Intravenous infusion of dopamine and normal saline were administered to maintain blood pressure because no other antiarrhythmic agents and defibrillator were available in the hospital. After blood pressure had returned to 90-100/50-60 mmHg, ventricular tachycardia persisted. Twelve and half hours later, the patient suddenly felt cold and chill, the intravenous infusion was immediately stopped, and 25 mg promethazine and 5 mg dexamethasone were intramuscularly injected at once. Thirty minutes later, the symptom was released. Heart rate reduced to 88 beats per min and the electrocardiogram revealed sinus rhythm.

Pyrogens were detected in the solutions, which caused the reaction in the patients.

## Discussion

Sustained ventricular tachycardia (SVT) can occur in patients with structurally normal and abnormal hearts, as did in case report 1 and case 2. Because SVT can lead to hemodynamic instability, it should be converted to sinus rhythm. Lidocaine is a common antiarrhythmic agent for treatment of SVT that does not cause hemodynamic decompensation. If lidocaine fails, amiodarone is another good choice for most cases without contraindication. If the arrhythmia does not respond to medical therapy, direct current cardioversion can be used. When SVT precipitates hypotension, shock, angina, congestive heart failure, or symptoms of cerebral hypoperfusion, it should be treated promptly with direct current cardioversion [2,3]. In the two patients, especially in cases 2, SVT failed to response to lidocaine should have been managed with direct current cardioversion, but because of no defibrillator, cardioversion could not be performed, which resulted in hypotension and shock. The conversion of SVT was due to pyrogen reaction.

Pyrogen reaction was frequently observed in the rural hospital in China during 1980s, when infusion equipment was reusable, because endotoxin, heat-stable denatured proteins, and other materials were difficult to remove from glass and rubber parts. Endotoxin, an exogenic pyrogen, can act on the thermoregulatory center located in anterior and posterior hypothalamus though leucocytic (endogenic) pyrogens, which shift upwards the thermostat setting in the hypothalamus. This results in signals from the posterior hypothalamus to increase heat production and decrease peripheral heat loss. Heat production from muscle contractions and heat conservation from peripheral vasoconstriction continue until the temperature of blood supplying the hypothalamus matches the higher thermostat setting and body temperature rises. So, pyrogen reaction is characteristically manifested by sudden cold, chill and fever during or after infusion, and lasts about half to one hour [1,4]-[6]. When the reaction happens, infusion should be stopped immediately, and antipyretic, antihistamine and steroids can be administered for releasing symptoms.

In this report, promethazine, an antihistamine, was used for treatment of pyrogen reaction in both patients. Perhaps promethazine had some relationship with the conversion of SVT. It has been reported that histamine could increase spontaneous rate and cause ventricular tachycardia in animal experiments [7,8]. But some second generation, not the first generation, antihistamines have also been reported to induce torsades de pointes ventricular tachycardia in human [9,10]. When used as a psychotropic drug, promethazine was reported to induce polymorphous ventricular tachycardia and torsades de pointes [11]. Therefore, it is hard to explain how promethazine converted the SVT to sinus rhythm.

## Conclusion

When the two patients with SVT failed to response to lidocaine which once converted the SVT and was in danger because of no other choice (without other antiarrhythmic drugs and defibrillator), the pyrogen reaction happened. After the reaction the SVTs were astonishingly converted to sinus rhythm. The conversion of sustained ventricular tachycardia might be related to pyrogen reaction. The phenomenon might imply a new therapy of SVT. The underlying mechanism is not clear and remains to be researched.

## Abbreviations

SVT: Sustained ventricular tachycardia.

## Consent

Written informed consents were obtained from the patient's relative for publication of this case report and accompanying images. A copy of the written consent is available for review by the Editor-in-Chief of this journal.

## Competing interests

The authors declare that they have no competing interests.

## Authors' contribution

QZF and XJM were involved in the patient care, acquisition of data, analysis and interpretation of data, QZF involved in review of literature, drafting and revising the manuscript. Authors have read and approved the final manuscript.
